# Bridging the gaps among research, policy and practice in ten low- and middle-income countries: Development and testing of a questionnaire for researchers

**DOI:** 10.1186/1478-4505-8-4

**Published:** 2010-01-29

**Authors:** David Cameron, John N Lavis, G Emmanuel Guindon, Tasleem Akhtar, Francisco Becerra Posada, Godwin D Ndossi, Boungnong Boupha, Research to Policy and Practice Study Team

**Affiliations:** 1 Centre for Health Economics and Policy Analysis, McMaster University, Hamilton, Ontario, Canada; 2 Department of Economics, McMaster University, Hamilton, Ontario, Canada; 3 McMaster Health Forum, Hamilton, Ontario, Canada; 4 Department of Clinical Epidemiology and Biostatistics, McMaster University, Hamilton, Ontario, Canada; 5 Department of Political Science, McMaster University, Hamilton, Ontario, Canada; 6 Pakistan Medical Research Council at Khyber Medical College, Pakistan; 7 Council on Health Research for Development, Mexico City, Mexico; 8 Tanzania Food and Nutrition Centre, Dar es Salaam, Tanzania; 9 National Institute of Public Health, Vientiane, Lao People's Democratic Republic; 10 Council of Medical Sciences, Ministry of Health, Vientiane, Lao People's Democratic Republic

## Abstract

**Background:**

A questionnaire could assist researchers, policymakers, and healthcare providers to describe and monitor changes in efforts to bridge the gaps among research, policy and practice. No questionnaire focused on researchers' engagement in bridging activities related to high-priority topics (or the potential correlates of their engagement) has been developed and tested in a range of low- and middle-income countries (LMICs).

**Methods:**

Country teams from ten LMICs (China, Ghana, India, Iran, Kazakhstan, Laos, Mexico, Pakistan, Senegal, and Tanzania) participated in the development and testing of a questionnaire. To assess reliability we calculated the internal consistency of items within each of the ten conceptual domains related to bridging activities (specifically Cronbach's alpha). To assess face and content validity we convened several teleconferences and a workshop. To assess construct validity we calculated the correlation between scales and counts (i.e., criterion measures) for the three countries that employed both and we calculated the correlation between different but theoretically related (i.e., convergent) measures for all countries.

**Results:**

Internal consistency (Cronbach's alpha) for sets of related items was very high, ranging from 0.89 (0.86-0.91) to 0.96 (0.95-0.97), suggesting some item redundancy. Both face and content validity were determined to be high. Assessments of construct validity using criterion-related measures showed statistically significant associations for related measures (with gammas ranging from 0.36 to 0.73). Assessments using convergent measures also showed significant associations (with gammas ranging from 0.30 to 0.50).

**Conclusions:**

While no direct comparison can be made to a comparable questionnaire, our findings do suggest a number of strengths of the questionnaire but also the need to reduce item redundancy and to test its capacity to monitor changes over time.

## Introduction

Describing efforts to bridge the gaps among research, policy and practice and monitoring changes over time in the nature and extent of engagement in bridging efforts could prove highly useful to those calling for and funding these efforts in low- and middle-income countries (LMICs), those supporting the strengthening of capacity to plan and undertake these efforts, and those seeking to learn from others engaged in particularly innovative efforts. The health ministers and heads of national delegations from 58 countries who participated in the Ministerial Summit on Health Research have good reason to want to know whether and how researchers, policymakers and healthcare providers are responding to their call to collaborate in efforts to bridge the gaps among research, policy and practice [1]. Similarly the World Health Assembly has good reason to want to know whether and how members states and WHO itself are responding to their call to develop mechanisms to bridge these gaps [2]. Questionnaires could assist those seeking to describe and monitor changes in researchers', policymakers', and healthcare providers' bridging efforts.

In this paper we describe how we developed and tested the questionnaire used in the first phase of a three-phase study in ten countries. This phase involved surveying researchers who were involved in the production of research on one of two health topics in six countries (China, Ghana, India, Laos, Mexico, and Senegal) and on one health topic in four countries (Iran, Kazakhstan, Pakistan, and Tanzania). The selected health topics, all of which are interventions that are well supported by systematic reviews, include: 1) insecticide-treated materials such as bed nets to prevent malaria;[3,4] 2) intrauterine devices as part of the contraceptive methods mix offered to expand women's and couples' family-planning choices; [5-7] 3) oral rehydration therapy to prevent dehydration in children with diarrhoea; [8-10] and 4) DOTS strategy to control tuberculosis[11-13]. We describe elsewhere the findings from our survey of researchers as well as the second phase of the study, which involved surveying healthcare providers whose practice related to one of the four topics [14-16].

While there have been many questionnaires developed to describe researchers' bridging activities in single high-income countries such as Canada [17-19], as well as to describe a select range of bridging activities of organizations such as guideline-producing organizations and health technology assessment (HTA) agencies [20], including one that included a range of LMICs [21], we are not aware of a questionnaire focused on researchers' engagement in bridging activities related to high-priority topics (and the potential correlates of their engagement) having being developed and tested in a range of LMICs.

## Methods

### Questionnaire development

#### Conceptual domains and question/item selection

We drew on the World Report on Knowledge for Better Health and four existing questionnaires to identify conceptual domains to be covered by the questionnaire [17,18,22-24]. One of the existing questionnaires had been piloted in four of the participating countries in 2002-2003 and the findings and their implications discussed at a workshop in June 2003. For the conceptual domains related to researchers' activities to bridge the gaps among research, policy and practice, which we call knowledge transfer and exchange (KTE) activities, we identified ten conceptual domains that can be grouped into three broad categories: 1) producer-push efforts (which includes what is "transferred" to target audiences outside the scholarly community; to whom and with what investments in fine-tuning the approach to them; by whom and with what investments in supporting their efforts; how, and specifically using what "passive" strategies, interactive strategies related to the research process, and interactive strategies outside the research process; and with what evaluations of the efforts); 2) efforts to facilitate user-pull (which includes both strategies that provide access to research and strategies that develop target audiences' capacity to use research); and 3) exchange efforts (which includes how target audiences are involved in research and KTE processes). For the conceptual domains related to potential system-level, organizational and individual correlates of researchers' engagement in bridging activities, we identified eight conceptual domains that can be grouped into four broad categories: 1) system-level correlates (which includes the state of research knowledge on the topic; barriers to and facilitators of KTE; and access to particular sources of information); 2) system-level and organizational correlates (which includes support for research and KTE both when respondents began conducting research on the topic and over the time that they conducted research on the topic); 3) organizational correlates (which includes support for KTE activities within the respondent's organization); and 4) individual correlates (which includes the nature of the respondent's own research and their views about who is responsible for undertaking KTE activities).

We also drew on the four existing questionnaires mentioned above to identify questions/items [17,18,23,24], supplemented these with questions/items that we developed ourselves, and solicited feedback on the draft list of potential questions/items from the ten country teams in early 2004. We distilled an initial pool of over 500 potential questions/items to 230 potential question/items before soliciting feedback and we distilled the pool of questions/items further to 152 questions/items based on the feedback we received. Of the 152 questions/items that we retained, 39 were drawn verbatim (or with only changes to the tense and/or the focus of on a single health topic) from existing questionnaires, 73 were based on questions/items found in these questionnaires, and 40 were created specifically for this questionnaire. (One team's published reliability assessment of an existing questionnaire had shown high internal consistency of items within several conceptual domains related to KTE activities with Cronbach's alphas greater than 0.77, and our own reliability assessment of an existing questionnaire had shown similarly high internal consistency with Cronbach's alphas > 0.75.)[17] We grouped items related to each of the 18 conceptual domains (and considered each group as a single question with multiple sub-questions) and grouped other items (such as demographics) into nine questions, for a total of 28 questions (and 152 specific items).

#### Response scales

We retained two five-point ordinal response scales for existing items and used the same scales for modified or new items. For questions addressing the frequency with which a respondent undertook KTE activities the scale was *never, rarely, occasionally, frequently*, and *always *(with each response category defined using examples in the introductory section of the questionnaire). For questions addressing views (i.e., potential system-level, organizational and individual correlates of engagement in KTE activities) the scale was *strongly disagree, disagree, neither agree nor disagree, agree*, and *strongly agree*. (Given that there may be cultural or other factors that influence whether the top response category is used, the survey data can likely best be presented as a proportion with the top two response categories combined.) For three countries we also measured frequency of engagement in specific KTE activities by asking the number of times during a typical 12-month period the respondent (or their organization) undertook the activities. For select other questions we used *yes/no/don't know *response categories, fill-in-the-blank responses or open-ended responses.

#### Translation

The original English-language questionnaire was used in Ghana, India, Pakistan, and Tanzania, WHO's expert translation service translated the questionnaire for China (Mandarin), Kazakhstan (Russian), Mexico (Spanish), and Senegal (French), and country teams translated the questionnaire for Iran (Persian) and Laos (Lao). We made minor wording changes to accommodate differences in local word use or to correct minor errors in translation, piloted the draft questionnaire in each country and among contacts in other countries, and corrected any minor wording issues identified during pilot testing. (An English-language version of the survey instrument is provided as Additional File 1.)

### Sample and questionnaire administration

We describe elsewhere our approach to identifying a sample of researchers and administering the questionnaire [14]. Briefly, we defined researchers as individuals who spent at least 10% of their time doing research, which includes the production, synthesis, and sharing of research. We contacted 544 researchers, received 368 completed questionnaires, for a response rate of 68%. Survey work was completed in all ten countries between April 2004 and April 2005. We used the 308 questionnaires completed by individuals who indicated that they undertook KTE activities (but not exclusively commercialization activities) in the hope that research on the topic would be considered and/or acted upon outside the scholarly community, for a response rate of 64% [(368-60)/(544-60)] among those who indicated that they met this criterion (Table 1).

**Table 1:** Response rates by country and health topic

Country	Insecticide-treated nets to prevent malaria	Intrauterine devices for contraception	Oral rehydration therapy to prevent dehydration in children with diarrhea	TB-DOTS to treat patients with tuberculosis	Total
China	-	25/25	-	25/36	50/61

Ghana	16/20	-	3/6	-	19/26

India	-	-	16/40	17/59	33/99

Iran	-	-	-	24/37	24/37

Kazakhstan	-	29/30	-	-	29/30

Laos	20/22	18/20	-	-	38/42

Mexico	-	22/50	-	26/40	48/90

Pakistan		-	13/37	-	13/37

Senegal	16/16	-	18/18	-	34/34

Tanzania	20/28	-	-	-	20/28

All	72/86	94/125	50/101	92/172	308/484

Note that the numerator includes those who completed the questionnaire and indicated in the questionnaire that they undertook KTE activities (but not exclusively commercialization activities) in the hope that research on the topic would be considered and/or acted upon outside the scholarly community. The denominator includes all researchers surveyed less those who completed the questionnaire and indicated that they did not undertake KTE activities in the hope that research on the topic would be considered and/or acted upon outside the scholarly community.

### Reliability

We calculated the internal consistency of items within each of the ten conceptual domains related to KTE activities and select conceptual domains related to potential correlates of these activities (specifically Cronbach's alpha) as a measure of the extent to which the questionnaire measures each of these conceptual domains in a reproducible manner [25]. We also calculated the proportion of respondents who provided the same ordinal scale response for each item within a conceptual domain (e.g., always chose the response ''frequently'' when asked about what is transferred) as an indirect check on whether the grouping of conceptually related items may have contributed to a pattern response. We did not conduct an exploratory factor analysis due to our relatively small sample size (N = 308) and we did not examine test-retest reliability.

### Validity

We convened several teleconferences with country teams and a workshop with country team representatives to assess whether, on the face of it, the questionnaire appeared to be measuring the desired conceptual domains (i.e., face validity) and to assess whether the questionnaire attempts to measure all of the relevant and important elements of complex conceptual domains that do not lend themselves to being measured directly (i.e., content validity). We assessed construct validity in two ways: 1) by calculating the correlation between scales and counts (i.e., criterion-related measures) for the three countries that measured frequency of engagement in specific KTE activities in both ways; and 2) by calculating the correlation between different but theoretically related (i.e., convergent) measures for all countries [26,27].

## Results

The internal consistency of items within each of the ten conceptual domains related to KTE activities was very high (with Cronbach's alphas ranging from 0.89 [0.86-0.91] for efforts to facilitate ''user pull'' using passive strategies to 0.96 [0.95-0.97] for efforts to evaluate KTE activities), indicating that the questionnaire measures each of these conceptual domains in a reproducible manner but with some degree of item redundancy (Table 2). The proportion of respondents who provided the same ordinal scale response for each item within a conceptual domain ranged from low (with proportions less than 20% for four of ten conceptual domains) to relatively high (with 35% of respondents providing the same ordinal scale response for all efforts to facilitate ''user pull'' using active strategies and 38% of respondents providing the same ordinal scale response for all push efforts that involved a fine-tuning of approach to specific target audiences), indicating that grouping of conceptually related items may have contributed to a pattern response and hence to higher than expected internal consistency of items within each conceptual domain. For some conceptual domains related to potential correlates of KTE activities, pattern response was extremely rare. For example, fewer than 1 in 100 respondents provided the same ordinal scale response for the conceptual domain related to views about the nature of their own research, which included both positively and negatively framed items.

**Table 2:** Internal consistency and pattern response

Conceptual domains	# of items	Cronbach's alpha (95% CI)	Proportion who provided the same ordinal scale response for each item within a conceptual domain
Push - What is "transferred" to target audiences outside the scholarly community (e.g., journal articles, systematic reviews, and messages that specified possible action)	7	0.89 (0.87, 0.91)	0.15

Push - To whom is research being transferred and with what investments in fine-tuning the approach to them (e.g., tailored the content of mailings or e-mails to specific target audiences)	7	0.90 (0.88, 0.91)	0.17

Push - By whom is research being transferred and with what investments in supporting their efforts (e.g., identified and worked with credible messengers)	8	0.91 (0.89, 0.92)	0.38

Push - How is research knowledge being transferred to particular target audiences, and specifically using what "passive" strategies (e.g., mailed or emailed brief summaries or messages without an explicit request)	15	0.93 (0.91, 0.94)	0.13

Push - How is research knowledge being transferred to particular target audiences, and specifically using interactions related to the research process (e.g., interacted when developing a specific research question, objectives or hypothesis)	7	0.94 (0.93, 0.95)	0.27

Push - How is research knowledge being transferred to particular audiences, and specifically using interactions outside the research process (e.g., interacted through events organized by them or their organization or through informal conversations)	10	0.90 (0.88, 0.92)	0.10

Push - With what efforts to evaluate their KTE activities (e.g., assessed any changes in their target audiences actual -- i.e., objectively measured -- behaviour that may be attributable to their KTE activities)	7	0.96 (0. 95, 0.97)	0.28

Facilitating "user pull" - What passive strategies have been used to facilitate user pull (e.g., provided access to a searchable database of articles, reports, syntheses or reviews on the topic)	5	0.89 (0.86, 0.91)	0.23

Facilitating "user pull" - What active strategies have been used to facilitate user pull (e.g., developed capacity of target audiences to acquire research on the topic through searchable databases)	4	0.91 (0.89, 0.93)	0.35

Exchange - What exchange efforts are undertaken (e.g., established or maintained long-term partnerships related to the topic with target audience representatives, involved target audience representatives in establishing the overall direction of their or their organization's research on the topic)	6	0.92 (0.91, 0.94)	0.26

The questionnaire-development process (specifically the use of previously tested items and several teleconferences with country teams to review both conceptual domains and specific items) yielded consensus before embarking on data collection that the questionnaire appeared to be measuring the desired conceptual domains (i.e., face validity) and attempted to measure all of the relevant and important elements of complex conceptual domains such as push efforts (i.e., content validity). The workshop with country team representatives yielded consensus after completing data collection that the questionnaire had both face and content validity. The only concerns raised at the workshop regarding face validity pertained to the use of specific terminology -- 1) systematic review even though it was defined in the questionnaire; 2) messages for researchers' target audiences that specified possible action even though three examples of alternative terminology were provided in the questionnaire; and 3) credible messenger even though it was defined in the questionnaire -- and both the accuracy of its translation and its comprehension by respondents, however, each concern was raised by at most two countries.

The correlation was moderate to high and consistently statistically significant between scales and counts (i.e., criterion-related measures) for the three countries that measured frequency of engagement in specific KTE activities in both ways, indicating good construct validity and the redundancy of having both types of measures in the questionnaire (Table 3). Eight of eleven pairs of items that measure the same KTE activity but in different ways showed high levels of association (gamma ≥ 0.50) and three pairs show moderate levels of association (gamma ≥ 0.30). The correlation was moderate and consistently statistically significant between different but theoretically related (i.e., convergent) measures for all countries (Table 4). One of five pairs of conceptually related items show a high level of association and four pairs show moderate levels of association.

**Table 3:** Assessment of construct validity using criterion-related measures

Question (and response set)	Item	Strength	Item	Question (and response set)
Please indicate how often you (and/or your organization working in conjunction with you or on your behalf) performed each of these knowledge transfer and exchange (KTE) activities related to the health topic: *Never; Rarely; Occasionally; Frequently; Always*	Provided reprints/copies of articles published in scientific journals to your target audiences (*not *including syntheses or formal systematic reviews of the research literature)	gamma = 0.626 p < 0.0005	Same as column 2	Please indicate how often during a *typical 12-month period *you (and/or your organization working in conjunction with you or on your behalf) performed each of these KTE activities related to the health topic: *Never; About 3-4 times during the last 12 months; About every month; Weekly; More than once a week*

	Provided syntheses of the research literature to your target audiences (*not *including formal systematic reviews of the research literature that follow explicit rules to reduce bias in searching the literature, identifying eligible articles, extracting data, etc.)	gamma = 0.648 p < 0.0005	Same as column 2	

	Provided formal systematic reviews of the research literature to your target audiences	gamma = 0.657 p < 0.0005	Same as column 2	

	Developed brief summaries of articles and/or research reports for your target audiences (*not *including brief summaries of syntheses and/or formal systematic reviews)	gamma = 0.478 p < 0.0005	Same as column 2	

	Developed messages for your target audiences that specified possible action	gamma = 0.452 p < 0.0005	Same as column 2	

	Developed reports, summaries or messages that provided examples or demonstrations of how specific target audiences could use the research	gamma = 0.364 p < 0.0005	Same as column 2	

	Mailed or e-mailed to your target audiences a *newsletter *containing brief summaries and/or messages	gamma = 0.629 p < 0.0005	Same as column 2	

	Accepted requests from journalists to participate in interviews or debates	gamma = 0.731 p < 0.0005	Accepted requests from journalists (radio, television, newspaper, etc.) to participate in interviews or debates	

Please indicate how often you (and/or your organization working in conjunction with you or on your behalf) interacted with representatives and/or members of your target audiences about research on the health topic in the following contexts *outside of the research process per se*: *Never; Rarely; Occasionally; Frequently; Always*	Interacted through government-sponsored meetings involving your target audiences	gamma = 0.692 p < 0.0005	Same as column 2	Please indicate how often during a *typical 12-month *period you (and/or your organization working in conjunction with you or on your behalf) interacted with representatives and/or members of your target audiences about research on the health topic in the following contexts *outside of the research process per se*: *Never; About 3-4 times during the last 12 months; About every month; Weekly; More than once a week*

	Interacted through events organized by you and/or your organization	gamma = 0.556 p < 0.0005	Same as column 2	

	Interacted through informal conversations with your target audiences	gamma = 0.556 p < 0.0005	Same as column 2	

**Table 4:** Assessment of construct validity using convergent measures

Question (and response set)	Item	Strength	Item	Question (and response set)
Please indicate how often you (and/or your organization working in conjunction with you or on your behalf) performed each of these knowledge transfer and exchange (KTE) activities related to the health topic: *Never; Rarely; Occasionally; Frequently; Always*	Provided reprints/copies of articles published in scientific journals to your target audiences (*not *including syntheses or formal systematic reviews of the research literature)	Gamma = 0.296 p = 0.008	Had access to at least five scientific journals indexed in *other *international reference databases (e.g., Medline, PubMed or the equivalent for your field) (full text paper or full text electronic)	Please indicate whether you had access to the following sources of information when you were involved in research on the health topic and in knowledge transfer and exchange (KTE) activities related to the health topic: *No; Yes*

	Same as above	gamma = 0.410 p < 0.0005	Had access to at least five scientific journals published locally, nationally or regionally (full text paper or full text electronic)	

	Provided formal systematic reviews of the research literature to your target audiences	gamma = 0.333 p = 0.006	Same as above	

Please indicate how often you (and/or your organization working in conjunction with you or on your behalf) interacted (e.g., through teleconferences or face to-face meetings involving a small number of people) with representatives and/or members of your target audiences *in each of the following stages of the research process *for all research projects related to the health topic with which you have been involved: *Never; Rarely; Occasionally; Frequently; Always*	Interacted when developing a specific research question, objectives or hypothesis	gamma = 0.502 p < 0.0005	Involved representatives and/or members of your target audiences in establishing the *overall direction of research *on the health topic conducted by you and/or your research organization	Please indicate how often you (and/or your organization working in conjunction with you or on your behalf) performed each of these knowledge transfer and exchange (KTE) activities related to the health topic: *Never; About 3-4 times during the last 12 months; About every month; Weekly; More than once a week*

Please indicate how often you (and/or your organization working in conjunction with you or on your behalf) performed activities (e.g., conducted workshops or seminars) to increase the capacity of your target audiences to use research on the health topic: *Never; Rarely; Occasionally; Frequently; Always*	Developed capacity of target audiences to *acquire *research on the health topic through searchable databases (e.g., MedLine, Cochrane, ... Index Medicus...., LILACS)	gamma = 0.398 p = 0.001	Had access to at least five scientific journals indexed in *other *international reference databases (e.g., Medline, PubMed or the equivalent for your field) (full text paper or full text electronic)	Please indicate whether you had access to the following sources of information when you were involved in research on the health topic and in knowledge transfer and exchange (KTE) activities related to the health topic: *No; Yes*

## Discussion

### Principal findings

We have developed a questionnaire focused on researchers' engagement in a broad range of activities to bridge the gaps among research, policy and practice on high-priority topics and the potential correlates of their engagement, translated the questionnaire into seven languages, and examined its reliability and validity in ten LMICs. The questionnaire can be modified to focus on different high-priority topics simply by changing the description of the topic in the introduction to the questionnaire because all subsequent questions refer in generic terms to "the health topic." The questionnaire's strengths are the high internal consistency within conceptual domains covered by the questionnaire and the questionnaire's face, content and construct validity. Its principal weaknesses are item redundancy and pattern response within select conceptual domains, which can be addressed by removing select items within each of the nine conceptual domains and also possibly by mixing items from different conceptual domains. In future administrations of the questionnaire, close attention should be paid to specific terminology, such as systematic reviews, messages, and credible messengers, to ensure a common level of comprehension across countries.

While no direct comparison can be made to another similarly focused questionnaire that has been developed and tested in a range of LMICs, the questionnaire-development process drew on four questionnaires developed to describe researchers' bridging activities in single high-income countries, two of which also showed high internal consistency of items within several conceptual domains related to KTE activities [17,18]. Our questionnaire aimed to be more comprehensive than these four questionnaires in both its breadth and depth, however, its depth appeared to come partially at the expense of some degree of item redundancy within select conceptual domains. The questionnaire can be used to describe and monitor changes in researchers', policymakers' and health care providers' bridging efforts.

### Strengths and limitations of the study

The strengths of the study include: 1) the breadth of countries engaged in developing and testing the questionnaire; 2) the mix of teleconferences before data collection and a workshop after data collection to assess face and content validity; and 3) the explicit examination of both internal consistency and construct validity (using both criterion and convergent testing). The limitations of the study include: 1) the inability to conduct an exploratory factor analysis due to our relatively small sample size; 2) the lack of test-retest reliability testing, which should be examined before the questionnaire is used to monitor changes over time in the nature and extent of KTE activities; and 3) the lack of an external measure of engagement in KTE activities (perhaps obtained through selective participant-observation or the use of other qualitative methods) that would have allowed us to examine the presence and magnitude of a social desirability bias within specific conceptual domains.

## Conclusions

The use of this questionnaire or a modified version of it could prove useful to those calling for and funding efforts to bridge the gaps among research, policy and practice in LMICs, those supporting the strengthening of capacity to plan and undertake these efforts, and those seeking to learn from others engaged in particularly innovative efforts. The additional testing of this or a modified version of the questionnaire should include test-retest reliability and comparisons against external measures.

## Competing interests statement

The authors declare that they have no competing interests.

## Authors' contributions

DC and JL: 1) contributed substantially to conception and design, acquisition of data, and analysis and interpretation of data, 2) drafted the article and revised it critically for important intellectual content, and 3) gave final approval of the version to be published. GEG, TA, FB-P, GN and BB: 1) contributed substantially to conception and design, acquisition of data, and/or analysis and interpretation of data, 2) provided feedback on the article; and 3) gave final approval of the version to be published. Members of the Research to Policy and Practice Study Team: 1) contributed substantially to acquisition of data; 2) reviewed drafts of the article; and 3) gave final approval of the version to be published.

## Supplementary Material

Additional file 1 **McMaster University/World Health Organization Questionnaire on Knowledge Transfer and Exchange in the Health Sector**. Questionnaire that was developed and tested. Click here for file
